# Evaluation of antibody responses against the whole virions of goatpox and sheeppox viruses after subcutaneous immunization of rabbits

**DOI:** 10.1016/j.heliyon.2022.e11745

**Published:** 2022-11-17

**Authors:** Takele Tesgera Hurisa, Guohua Chen, Huaijie Jia, Fang Yong Xiang, Xiao-Bing He, Zhi-Zhong Jing

**Affiliations:** State Key Laboratory of Veterinary Etiological Biology, Key Laboratory of Veterinary Public Health of Agriculture Ministry, Lanzhou Veterinary Research Institute, Chinese Academy of Agricultural Sciences, Lanzhou 730046, Gansu, China

**Keywords:** Goatpox virus, Polyclonal antibody, Rabbit, Sheeppox virus

## Abstract

Antibody development is the integral process of generating and characterizing an antibody. It commences by inoculating the antigen of interest into laboratory animals, allowing the immune system develops large quantities of antibodies. This was aimed at developing antibodies against the virion of Goatpox and Sheeppox virus vaccines. The ability of Goatpox and Sheeppox vaccines was assessed. Regarding this study, the antibody titers against both Goatpox and Sheeppox viruses was increased in the same manner. The amount of IgG was determined to be 2.29 μg/μl and 2.18 μg/μl against virions of Goatpox virus and Sheeppox respectively. The purified IgG was analyzed by SDS-PAGE. Different bands of the purified antibodies were clearly visualized, and the molecular weight of IgG was estimated to be 67 kDa and 25 kDa. Additionally, antigen/antibody binding was confirmed by Western blot using GTPV A27 antigen. No significant differences in antibody titers were observed between the two groups (p < 0, 05).

## 1 Introduction

Purified antibodies have a fundamental role in the diagnosis and treatment of infectious diseases. Considering the progress in biotechnology and the production of recombinant drugs, antibodies are used to separate and recognize manufactured products. Polyclonal antibodies are a set of diverse immunoglobulin molecules produced against a specific antigen by different B cell lineages within the body in which each of them identifies the epitope. The polyclonal B cell response is a natural mode of the immune response exhibited by the adaptive immune system of mammals [7, 12]. Each arm of the immune system plays a crucial role in the recognition and elimination of poxvirus infection. Within a short period, enough humoral poxvirus-specific antibodies can develop and destroy the disease-causing virus. During the removal of infection, antibodies use a variety of mechanisms, such as virus neutralization and complement activation, opsonization, and antibody-dependent cellular cytotoxicity (ADCC) [8]. To date, there have been insufficient experimental studies on the production of polyclonal antibodies against the virion of the Goatpox virus (GTPV) and Sheeppox virus (SPPV). The main aim of the current study was to produce and compare antibody titers obtained in rabbits sera against Goatpox and Sheeppox viruses and to purify IgG. The whole virion of Goatpox and Sheeppox viruses was inactivated and emulsified by Freund’s complete adjuvant and inoculated subcutaneously at multiple sites, followed by a booster using Freund’s incomplete adjuvant. We conducted antibody purification using the ammonium sulfate precipitation technique, followed later by dialysis against 5 L of PBS overnight at 4 °C [6]. The purified antibody was quantified for protein concentration and tested by SDS-PAGE and Western blot [5].

## 2 Materials and methods

### 2.1 Production of polyclonal antibodies

Goatpox virus (strain AV41) and Sheeppox virus (strain AV42) were propagated using Vero cells obtained from ATCC (CCL81) in Eagle’s minimum essential medium (EMEM) with the addition of 2 % bovine calf serum (BCS). The infected cells were harvested when 80 % of the cells showed cytopathic effects. The viruses were concentrated and purified by high-speed centrifugation, passed through a 10–50 % sucrose density gradient, and resuspended in phosphate-buffered saline. The purified virus was treated with 1 % formalin at +4 °C, for 1 week for inactivation. The vaccine was prepared in the laboratory after equal volumes of each virus suspension were mixed with the adjuvant.

Six genetically approved New Zealand female white rabbits were purchased from the Lanzhou Veterinary Research Institute laboratory animal breeding section. The rabbits were immunized subcutaneously at multiple sites with 2 ml of inactivated Goatpox and Sheeppox viruses containing 0.5 mg/ml with Freund’s complete adjuvant (Sigma, ALDRICH, USA, Lot No SLBV6904). We carried out subsequent boosters at 14-day intervals with the same preparation of Freund’s incomplete adjuvant. Before each immunization, blood was drawn by venous puncture from the marginal ear vein and allowed to clot for 3 h at room temperature before the sera were collected. The titration of the specific polyclonal antibodies was performed 1–10 days after each boost. The serum dilution, the working dilution of secondary antibodies, and the reaction time was optimized using serum obtained from naturally infected animals by Goatpox and Sheeppox viruses. And then the experimental serum obtained from the rabbits was titered by ELISA.

The ELISA cut-off value was decided based on the method reported by Kumar and Rao (1991), using the mean absorbance of negative control plus three times the standard deviation and the antibody titer determined. Once the obtained antibody titer was satisfactory, whole blood was collected, and rabbits were killed by cervical dislocation under anesthesia. Subsequently, the mean OD values of the positive and negative results were analyzed by Microsoft Excel Windows 10. Antibody purification was conducted by the ammonium sulfate precipitation method [6]. The result was confirmed by SDS-PAGE and Western blot. Preimmune withdrawn blood was used as a negative control. We observed no local reaction or abnormalities at the site of injection during the experiment.

### 2.2 Ethics statement

The responsible governmental authorities registered and approved the use of rabbits during the experiment (Office for Health and Social Affairs in Lanzhou Veterinary Research Institute; license number SYK2015-0003) and they were housed according to national regulations. We monitored the physical conditions of the animals daily. We supplied the animals with feed and enough water. No animal was ill or died during the period of the experiment. Blood from the rabbit was collected aseptically. We made every endeavor to minimize pain and distress during the production of polyclonal antibodies following established best practices [2].

### 2.3 Determination of antibody titers

The blood was collected and the antibody titers of the sera were measured using the enzyme-linked immunosorbent assay (I-ELISA) as demonstrated previously by Vogt et al (23). Briefly, 96 wells of flat bottomed microtiter plates (Nunc, Beckton-Dickinson, NJ) were coated with 0.5 μg/μl of the purified A27R recombinant protein in coating buffer (100μl/well) and incubated overnight for 14 h at +4 °C then washed three times by PBS containing 0.05 % tween 20 (PBST). After washing, 200 μl of 1% BSA (Bovine Serum Albumin) fraction V (Roche, Germany) in PBS was added for blocking and incubated for 2 h at 37 °C. The collected serum was diluted serially in each well and again incubated for 1 h at 37 °C. After washing steps mouse anti-rabbit IgG conjugated with HRP was diluted in blocking buffer in the ratio of 1:8000 (100μl/well) and added to each well. Following the incubation and washing process, 100 μl of the substrate (55 mM 3, 3′, 5, 5′- tetramethylbenzidine, 0.03% H2O2) was added to each well and again incubated at 37 °C for 15 min. The reaction was stopped by 50 μl of 2M H2SO4, and the OD value was read at 450 nm using the spectrophotometer (24). The antibody endpoint titer was determined and defined as the reciprocal of the dilution resulting in an absorbance value that was twice that of the background.

### 2.4 Purification of IgG

An ammonium sulfate precipitation method was carried out for IgG purification. We diluted rabbit serum at a proportion of 1:2 with PBS on ice. Saturated 45 % (v/v) ammonium sulfate was added to the final concentration in a dropping manner, and the mixture was stirred at +4 °C for 30 min and centrifuged at 1000 × g for 15 min. The precipitate was collected and washed with 45 % saturated ammonium sulfate, centrifuged again at 1000 × g for 15 min, redissolved in the same volume of PBS as the original antibody solution, and centrifuged again at 5000 × g for 15 min to remove any insoluble material. The supernatant was transferred to clean tubes, and the immunoglobulin was reprecipitated by using 40 % saturated ammonium sulfate. The centrifugation was repeated at 1000 × g for 15 min. The precipitate was again redissolved in PBS (5 ml) and dialyzed against 5 L of PBS at +4 °C overnight to remove trace amounts of ammonium ions to prevent interference from subsequent procedures, such as conjugation of antibodies with biotin and fluorochromes or agarose, and centrifuged at 5000 × g for 15 min to remove more insoluble substances. Finally, the supernatants were collected, and an aliquot was examined by SDS-PAGE; the rest were stored at -70 °C [6].

### 2.5 Western blot

In this study, GTPV A27 protein with a molecular weight of 35 kDa was used for blotting. The protein band obtained by SDS-PAGE was transferred to a nitrocellulose membrane and then probed with the corresponding antibody. Western blotting or immune blotting was used to detect the target protein [6].

### 2.6 Statistical analysis

The statistical analysis of OD values of antibody titration was performed by Microsoft Excel Windows 10.

### 2.7 Compliance with the guidelines

In this study, the experimental design, sample size, inclusion and exclusion criteria, randomization, blinding, outcome measurements, statistical methods, experimental animals, and procedures, and the results were carried out according to the compliance of guidelines for Ethical Conduct in the Care and Use of Nonhuman Animals in Research.

## 3 Results

**Figure 1:**
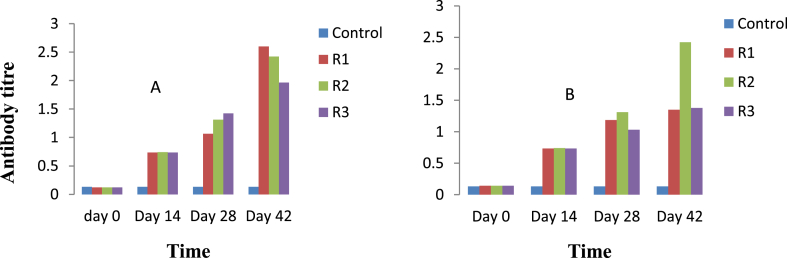
Antibody titers in the serum of immunized rabbits. For both groups, the rabbits (n = 3) were immunized with inactivated virion of Goatpox virus (1A) and Sheeppox virus (1B) vaccines 3 times at an interval of 2 weeks by subcutaneous (SC) route. The titer in each sample was determined by indirect ELISA, and geometric mean titers were calculated and plotted against time. The antibody titer was expressed as the end-point sample dilution. Each bar represents the geometric mean antibody titer. No difference was shown in antibody titer between the groups except in the individual rabbits that revealed on day 42. Whereas a significant difference was witnessed between the control and immunized rabbits in both groups (P˂ 0.05).

**Figure 2:**
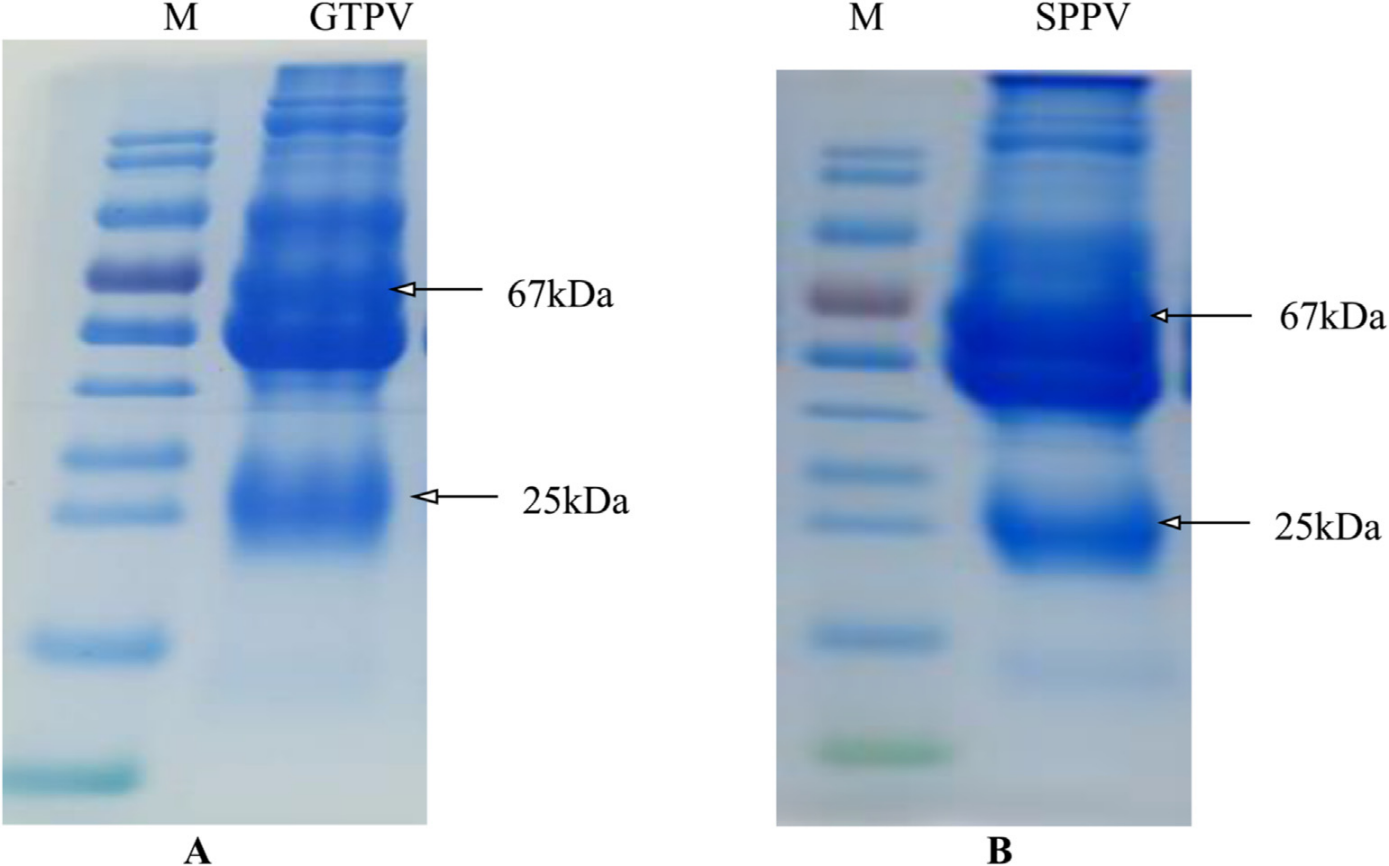
SDS-PAGE analysis of rabbits IgG against the whole virion of Goatpox virus (2A) and Sheeppox virus (2B). Based on the SDS-PAGE analysis a *Capripoxvirus* specific 67 kDa heavy chain and 25 kDa light chains were detected. A prominent non specific band was also detected against both vaccines under the 67KDa.

**Figure 3:**
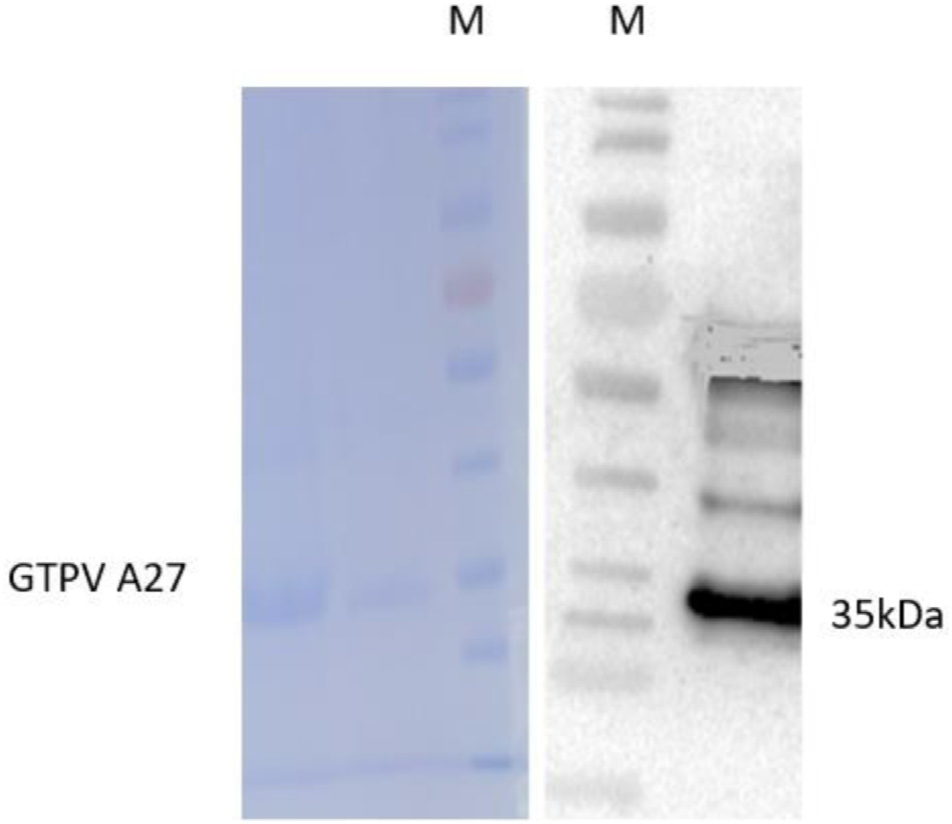
Antigen/antibody binding was checked by Western blot. A distinguished band was observed at 35 kDa, which is the molecular weight of GPV A27.

A virus neutralization titer is currently considered as the gold standard for detecting and measuring antibodies that can neutralize viruses causing many diseases. This preliminary work was intended to produce polyclonal antibodies against the virion of GTPV and SPPV in rabbits. Hence, rabbits were immunized, and antibodies were collected and purified. The mean OD values of positive and negative results were analyzed by Microsoft Excel for Windows 10. The antibody titers increased in a similar manner, with some deviation among individual rabbits [Figure 1(A, B)]. The amounts of IgG was determined to be 2.29 μg/μl and 2.18 μg/μl against virions of GTPV and SPPV, respectively. The purified IgG was analyzed by SDS-PAGE with Page RulerTM Prestained Protein Ladder parts No. 26616 and 26617 standard molecular protein markers [Figure 2(A, B)] for the virions of GTPV and SPPV, respectively. When the bands were compared, the IgG bands against virions of the sheeppox were slightly more prominent. We estimated the size of different bands of antibodies to be 67 kDa and 25 kDa regarding both GTPV and SPPV vaccines. Additionally, antigen/antibody binding was confirmed by Western blotting with the GTPV A27 antigen (Figure 3). We observed no significant differences in antibody titers between the two groups (p ˂ 0.05).

## 4 Discussions

In biomedical studies, antibodies are emerging as fundamental tools with great commercial and medical value. Also, antibodies are considered the fastest growing product sector of the pharmaceutical industry. Therapeutic antibodies are significant drugs to treat autoimmune diseases and infections. Furthermore, antibodies are important substances in diagnostic and medical research, including therapeutic effects [1, 4]. For instance, they are a combination of monoclonal antibodies (Abs) produced against different epitopes of antigens and have great avidity to polyvalent antigens [11, 12]. In the present study, we produced and evaluated the antibody response against the virion of GTPV and SPPV vaccines after subcutaneous immunization of rabbits. A similar antibody titer was observed against both GTPV and SPPV vaccines, with little variation between individual rabbits. During SDS-PAGE analysis, IgG of 67 kDa and 25 kDa were detected in both groups. Related studies showed that immunization with Goatpox and Sheeppox antigens was started in 1903 by Borrel [21].

Previous investigations illustrated that *capripoxvirus* has five major structural proteins. Based on the Western blot analysis of virus-specific antibody responses to *Capripoxvirus* and contagious pustular dermatitis infections in sheep, Chand et al. (14) described the major proteins, including polypeptides of different molecular weights of *Capripoxvirus*. The 67 kDa and 32 kDa on the envelope of the virus have unlike functions in which the former can precipitate antigens, such as the 45 kDa protein, and contains cross-reactive antigenic determinants [14]. The 32 kDa protein contains epitopes that induce neutralizing antibodies, signifying that the protein is strongly immunogenic [10]. Chand et al. (14) also mentioned the properties of the proteins 26 kDa and 19 kDa in the virion's core of *Capripoxvirus*, where 26 kDa can induce neutralizing antibodies. Sheeppox, Goatpox, and lumpy skin disease viruses are similar, sharing 97 % nucleotide identity, and studies have also revealed that Sheeppox and Goatpox viruses share approximately 96 % nucleotide identity over their entire length [14]. Taking into consideration the genomic resemblance of *Capripoxviruses*, we used the GTPV A27 antigen to determine the antibody titer against Goatpox and Sheeppox viruses in the blood of immunized rabbits, and we observed almost similarities in positive and negative OD values in both virus groups that share similar nucleotides [Figure 1(A, B)]. This was further strengthened by the absence of significant differences in the antibody titer values for virions of the two poxviruses. When the 67 kDa heavy and 25 kDa light chains of IgG were compared, the bands produced against the Sheeppox virus were slightly more prominent [Figure 2(A, B)]. In this study, the protein concentration was estimated spectrophotometrically at 280 nm. Stern D et al (2016), reported that the surface protein A27 was found to be a well-bound, highly immunogenic, and exposed target for antibodies aiming at virus particle detection [18]. Another report by Thomas Kaever et al (2016), described that inoculation with vaccinia virus (VACV) elicits neutralizing antibodies against major antigens, including A27, A33, B5, D8, H3, and L1 on both the extracellular enveloped virus (EV) and the intracellular mature virion (MV or IMV), conferring protection against smallpox [19]. Additionally, Hurisa et al (2021), have compared the immunogenicity of three Capripoxvirus proteins; GTPV A 27R, GTPV L1R, and SPPV A 33R proteins, and reported that GTPV A 27R protein showed a better detection ability and can be used for the detection of antibodies produced against *Capripoxvirus* in sera of animals [22]. Furthermore, M. Dashprakash et al (2019), reported the diagnostic efficacy of rA27L of GTPV in an indirect ELISA and showed its high specificity, as it was highly reactive with anti-GTPV and anti-SPPV sera but not with antisera against other related viruses of sheep and goats [25]. A similar report by Kumar A *et al* (2015), indicated that the diagnostic efficacy of rA27L of buffalopox virus (BPXV) in indirect-ELISA showed high specificity as it was highly reactive with anti- Bufallopox virus (BPXV) and anti-Camelpox virus (CMLV) sera coupled with a negligible reactivity was observed against viruses from other genera [26].

Therefore, based on these facts, the GTPV A27 antigen, which was prepared in-house, was purified and optimized, and used for evaluation of the titer of antibodies. No significant difference was observed between the two *Capripoxvirus* vaccines.

## 5 Conclusions

The virion of the Goatpox and Sheeppox virus vaccines stimulated enough IgG. The observed 67 kDa represents the heavy chain, while the 25 kDa is the light chain. The presented results suggest that immunization using Goatpox and Sheeppox virus vaccines can stimulate adequate antibodies in rabbits.

### 5.1 Ethics statement

All animal experiments followed the regulations of the Institutional Animal Care and Use Committee of Lanzhou Veterinary Research Institute (license number SYK2015-0003).

### 5.2 Statistical analysis

Microsoft Excel Windo 10 was employed for statistical analysis.

## Declarations

### Author contribution statement

Zhi-Zhong Jing; Takele Tesgera Hurisa: Conceived and designed the experiments.

Takele Tesgera Hurisa: Performed the experiments; Analyzed and interpreted the data; Wrote the paper.

Guohua Chen; Huaijie Jia: Performed the experiments.

Zhi-Zhong Jing; Fang Yong Xiang; Xiao-Bing He: Contributed reagents, materials, analysis tools, or data.

### Funding statement

This work was supported by National Science and Technology Support Program of China.

### Data availability statement

Data will be made available on request.

### Declaration of interest’s statement

The authors declare no conflict of interest.

### Additional information

No additional information is available for this paper.

## Appendix A Supplementary data

Suplementry materials Suplementry materials
