# Supplementary material for: Evaluation of antibody responses against the whole virions of goatpox and sheeppox viruses after subcutaneous immunization of rabbits
Source: Heliyon. 2022 Nov 17;8(11):e11745. doi: 10.1016/j.heliyon.2022.e11745 (PMC9681629; doi:10.1016/j.heliyon.2022.e11745)
Supplement: Suplementry materials [file mmc1.docx]

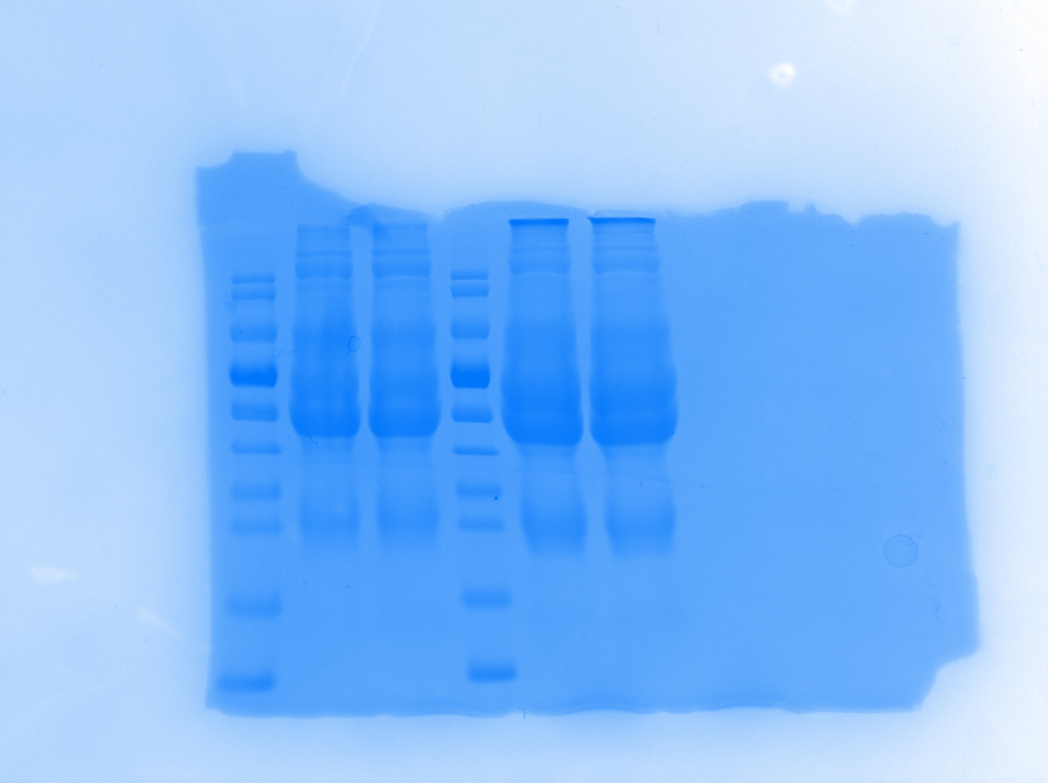


67 kDa

25 kDa

67 kDa

25 kDa

SPPV

M

M

GTPV

Figure 2. SDS-PAGE result of antibody against GTPV and SPPV

M


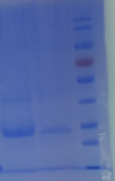


35 kDa GTPV A 27 protein

Figure 3. SDS-PAGE result of GTPV A27 protein

M


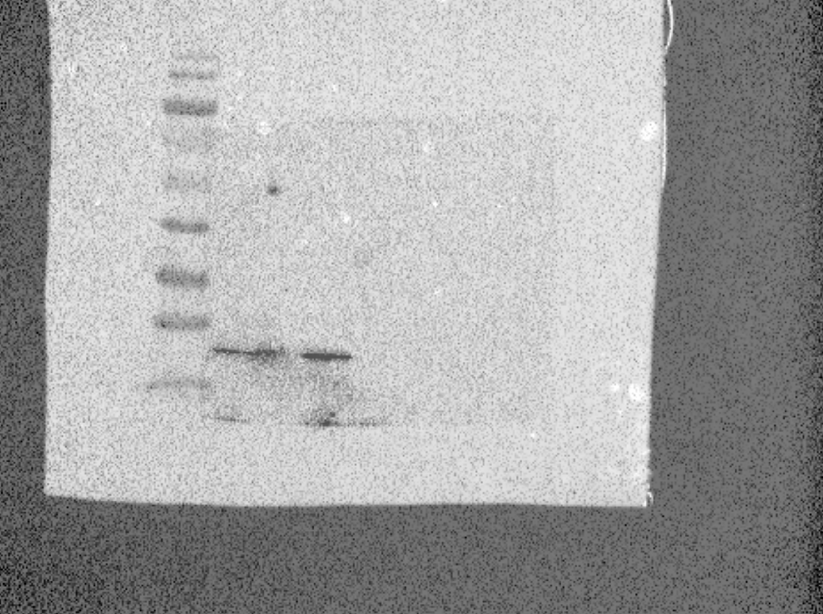


Ag/Ab binding at 37 kDa

3. Western blot analysis result
